# Combined Effects of Nitrogen Concentration and Seasonal Changes on the Production of Lipids in *Nannochloropsis oculata*

**DOI:** 10.3390/md12041891

**Published:** 2014-03-31

**Authors:** Martin Olofsson, Teresa Lamela, Emmelie Nilsson, Jean-Pascal Bergé, Victória del Pino, Pauliina Uronen, Catherine Legrand

**Affiliations:** 1 Faculty of Health and Life Sciences, Centre for Ecology and Evolution in Microbial Model Systems (EEMiS), Linnæus University, 391 82 Kalmar, Sweden; E-Mails: martin.olofsson@lnu.se (M.O.); emmelie.nilsson@lnu.se (E.N.); 2 Necton SA, Belamandil s/n, 8700-152 Olhão, Portugal; E-Mails: teresa@necton.pt (T.L.); vdelpino@necton.pt (V.P.); 3 IFREMER, Laboratoire de Science et Technologie de la Biomasse Marine (STBM), 44311 Nantes cedex 03, France; E-Mail: jpberge@ifremer.fr; 4 Neste Oil, Technology Centre, POB 310, 06101 Porvoo, Finland; E-Mail: pauliina.uronen@nesteoil.com

**Keywords:** microalgae, outdoor, *Nannochloropsis*, large-scale, lipids, fatty acids, nitrogen manipulation, seasonal changes, biofuels, high value products

## Abstract

Instead of sole nutrient starvation to boost algal lipid production, we addressed nutrient limitation at two different seasons (autumn and spring) during outdoor cultivation in flat panel photobioreactors. Lipid accumulation, biomass and lipid productivity and changes in fatty acid composition of *Nannochloropsis oculata* were investigated under nitrogen (N) limitation (nitrate:phosphate N:P 5, N:P 2.5 molar ratio). *N. oculata* was able to maintain a high biomass productivity under N-limitation compared to N-sufficiency (N:P 20) at both seasons, which in spring resulted in nearly double lipid productivity under N-limited conditions (0.21 g L^−1^ day^−1^) compared to N-sufficiency (0.11 g L^−1^ day^−1^). Saturated and monounsaturated fatty acids increased from 76% to nearly 90% of total fatty acids in N-limited cultures. Higher biomass and lipid productivity in spring could, partly, be explained by higher irradiance, partly by greater harvesting rate (~30%). Our results indicate the potential for the production of algal high value products (*i.e.*, polyunsaturated fatty acids) during both N-sufficiency and N-limitation. To meet the sustainability challenges of algal biomass production, we propose a dual-system process: Closed photobioreactors producing biomass for high value products and inoculum for larger raceway ponds recycling waste/exhaust streams to produce bulk chemicals for fuel, feed and industrial material.

## 1. Introduction

Microalgae have been proposed as feedstock for biodiesel due to their rapid growth and high lipid content [1,2,3]. The advantages with microalgae derived biodiesel may be (1) higher biomass productivity compared to land grown crops, (2) possibility to grow on marginal or non-arable land, (3) utilization of seawater and waste water, therefore reducing fresh water use, (4) both CO_2_ neutral fuel manufacture and CO_2_ sequestration (5) non-toxic, biodegradable and renewable fuel [1,2,4,5]. However, Tredici [6] argues that microalgae are not superior to land grown agro crops in terms of photosynthetic efficiency and biomass productivity. The main advantage would rather be the ability of microalgae to alter their cellular composition as a response to distinctive culture conditions (*i.e*., nutrient deficiency). Thus, the potential lipid production of microalgae is much greater than agro crops.

Few microalgal species accumulate large quantities of lipids during exponential phase and the lipids are primarily present as structural polar lipids, typically polyunsaturated fatty acids (PUFA), which can be commercially valuable as food supplement [7] and for nutritional enrichment in aquaculture industry [8,9,10]. In particular, the genus *Nannochloropsis* contains high amounts of eicosapentaenoic acid (20:5 ω3, EPA) [11,12,13]. EPA can serve as a marine drug since it has a well documented positive effect on human health [14]. At stationary phase microalgae can accumulate substantial amounts of neutral storage lipids in the form of triglycerides (TAGs), which are considered as the best substrate for biodiesel [15,16]. TAGs consist principally of saturated fatty acids (SAFA) and monounsaturated fatty acids (MUFA). Biodiesel is manufactured using a transesterification process where vegetable or animal TAGs are reacting with an alcohol (typically methanol) to produce an ester then referred to as a fatty acid methyl ester (FAME) and glycerol [1]. Distinct from biodiesel is renewable diesel, such as hydrotreated vegetable oil (HVO) that is chemically similar to petrodiesel, but is derived from a wide range of vegetable sources, although waste animal fats and other waste and residue streams can be used. In the process hydrogen is used to remove oxygen from TAGs, which produces a pure hydrocarbon chain (paraffin) containing no oxygen but small amounts of water, CO_2_ and propane as by-products [17].

Nutrient stress, mainly nitrogen (N) limitation or deprivation, is well known to enhance lipid accumulation in microalgal cells with generally higher degrees of SAFA and MUFA [18,19,20,21,22]. Other growth conditions affecting lipid content and composition are CO_2_ concentration [23,24], light [8,11,12,25,26], temperature [25,26,27,28,29,30], salinity [31,32] and growth phase [33,34]. Increased lipid content in microalgal cells due to nutrient stress induction may not always yield a net gain in algal oil since biomass production usually is reduced. There are examples though, of net gain in lipid productivity in large-scale cultures [21,35] exposed to complete nutrient starvation during high productive seasons. Whether this net gain in lipid production can be sustainable over the entire production season is unknown. Thus, we investigated the combined effect of nutrient limitation and seasonal variation (autumn and spring) on algal lipid production using *Nannochloropsis oculata*.

In contrast to the numerous laboratory-based experiments, nutrient stress induced algal oil production needs to be demonstrated in scaled-up conditions outdoors. Thus, outdoor large-scale cultures of *N. oculata* grown in flat panel photobioreactors (PBRs) were nutrient manipulated at steady-state to maximize algal lipid production in *N. oculata*. Total lipids (TL), fatty acid (FA) profiles, biomass productivity (BP) and lipid productivity (LP) were determined during the study. The aim was to increase lipid content and change FA composition through nutrient limitation without substantial loss of biomass resulting in a total net gain in lipid productivity. The study was conducted with N-manipulation experiments in autumn 2008 and in spring 2009. Seasonal conditions can be specific to geographical location and may vary among years. As seasonal variations cannot be manipulated, it is important to include their impact in combination with nitrogen stress on algal productivity to improve estimates of lipid productivity over an entire year.

## 2. Results and Discussion

Ambient daily average temperatures ranged 19–21 °C during autumn 2008 and 17–20 °C during spring 2009. Total global radiation (TGR) in autumn 2008 varied between 15 and 20 MJ m^−2^ day^−1^ with a dip as low as 5 MJ m^−2^ day^−1^ on day 4 during the first experiment. TGR in spring 2009 was higher and ranged 23–31 MJ m^−2^ day^−1^.

### 2.1. Optical Density (OD) and Dry Weight (DW)

**Figure 1:**
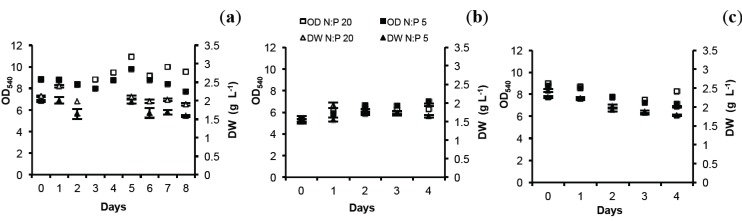
Biomass of *N. oculata* illustrated by optical density at 540 nm (OD_540_) and dry weight (DW) at different N-limitations. Autumn: (**a**) N:P 5. Spring: (**b**) N:P 5; (**c**) N:P 2.5. DW shows mean values ± SD of 3 technical triplicates.

Both in autumn and spring, algal biomass followed a similar pattern in control (N:P 20, molar ratio) and N-limited cultures (N:P 5, N:P 2.5), regardless of N-limitation level (Figure 1). Significantly lower biomass (DW) was found in N-limited cultures compared to control in both autumn and spring at N:P 5(ANOVA, *p* = 0.00732). At N:P 2.5 in spring, there was no difference between treatment and control indicating very little effect of the level of N stress(Fisher test, *p* = 0.5993). DW showed no lag phase in response to N-limitation compared to OD. This trend was clearer in the autumn experiment as the study period was 9 days instead of 5 days (spring).

### 2.2. Total Lipids (TL) and Total Protein (TP)

**Figure 2:**
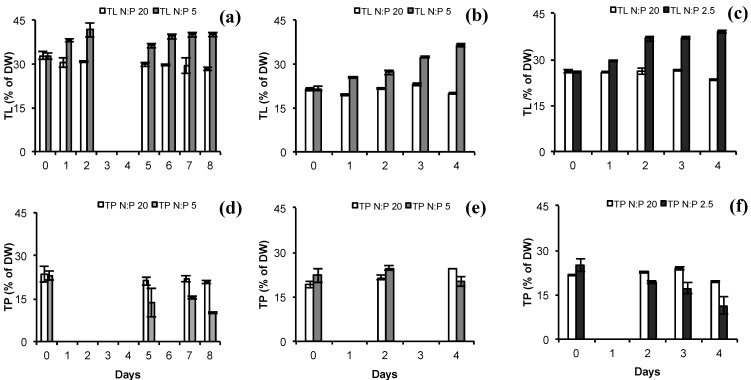
Total lipids (TL) and protein (TP) total in % of dry weight for *N. oculata* at different N-limitations. (**a**) TL at N:P 5 in autumn; (**b**) TL at N:P 5 in spring; (**c**) TL at N:P 2.5 in spring; (**d**) TP at N:P 5 in autumn; (**e**) TP at N:P 5 in spring; **(f**) TP at N:P 2.5 in spring. White bars represent control (N:P 20) for both TL and TP, light grey bars represent N:P 5 values for both TL and TP, and dark grey bars represent N:P 2.5 for both TL and TP.TL and TP show mean values ± SD of 5 and 3 technical replicates, respectively.

There was a significant effect of treatment (ANCOVA, *p* = 8.89 × 10^−7^), season (ANCOVA, *p* = 7.92 × 10^−8^) and an interaction effect of time and treatment on TL (ANCOVA *p* = 0.0121). N-limited cultures accumulated more lipids compared to control and the slope (the rate of increase in lipid accumulation) was higher in autumn. TL concentrations in % of DW for *N. oculata* showed an increase with 10–15 percentage points compared to control in 48 to 96 h by N-stress (Figure 2d–f). The TL reached 40% in both autumn (N:P 5) and spring (N:P 2.5). Lipid accumulation for microalgae at N-deficiency or N-limitation has been reported by several studies [18,19,20,21,22,35,36,37] and the conditions applied by these studies vary widely, making comparison difficult. Moreover, the actual increase in TL may be species and strain specific. [21,35,38,39,40,41,42]. Our results of TL were in consent with literature, although the increase was not as drastic as for outdoor studies of *Nannochloropsis* sp. F&M-M24 strain [21,35] and *Neochloris oleoabundans* [22]. Both species doubled the TL content up to 50%–60% of DW after N-starvation. The N-stress level in spring (N:P 2.5 *vs.* N:P 5) did not have a major effect on the amount of lipids accumulated and the differences in percentage points between treatment and control were similar (16%). However, a significant interaction effect of both N:P 5 and N:P 2.5 and time on lipid accumulation was found (ANCOVA, *p* = 0.0011 and *p* = 0.00174, respectively).

Nitrogen is essential for protein synthesis [43]. Hence protein content may be reduced at nitrogen limitation [36,44], which also was a trend for *N. oculata* in the N-limited treatments compared to the controls (Figure 2a–c). A significant interaction effect of time and treatment was found (ANCOVA, *p* = 0.0065) and a seasonal effect (ANCOVA, *p* = 0.0305). The TP content decreased from approximately 20% of DW at N-sufficient growth to nearly half the protein content observed at N-limitation (N:P 5 in autumn and N:P 2.5 in spring). The use of co-products in algal biofuels production must take into account the lower protein content at N-limitation or N-deprivation. TP content for control cultures was similar among autumn and spring experiments (20% of DW).

The trend for the total lipid to total protein (TL/TP) ratio implied an increase at N-stress with average ratios of 2 at N-limitation compared to 1.2 for control cultures (data not shown). Killham *et al.* [45] reported similar relative values for the freshwater green algae *Ankistrodesmus falcatus*. However, the values in spring at N:P 5 were similar to the control as shown by only minor decrease in TP (Figure 2b).

### 2.3. Fatty Acid (FA) Profiles

Fatty acid (FA) profiles of *N. oculata* were comparable to those of other *Nannochloropsis* strains [8,11,12,21,22,26,35,46,47,48]. In this study, FA profiles of *N. oculata* under N-limitation differed mainly in respect of C16:0, C18:1 and C20:5. N-stressed cells accumulated more C16:0 and C18:1 but less C20:5 compared to control (Table 1). Minor differences could also be observed for C14:0, C18:2 (spring) and C20:4. The level of N-stress (N:P 2.5 *vs.* N:P 5) seemed to have a stronger influence on the profile compared to the length of the N-stress. In spring, the difference in C16:0 was as much as 15 percentage points and the discrepancy in C20:5 was 10 percentage points at the most severe N-stress (N:P 2.5) compared to 10 and 8 percentage points (N:P 5), respectively. The profile after 8 days of N-stress was similar to the profile after 4–5 days (N:P 5).

**Figure 3:**
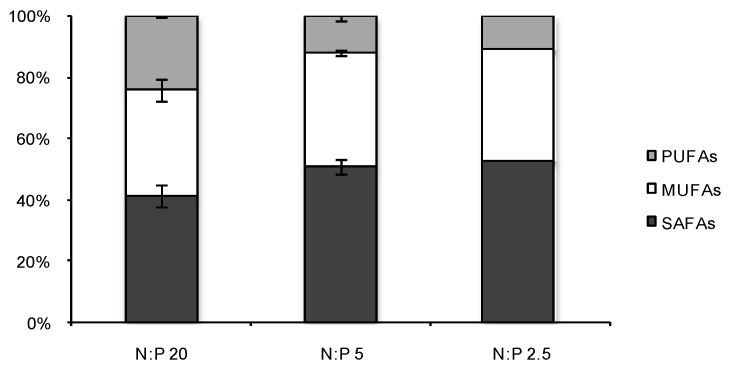
Fractions of saturated fatty acids (SAFAs), monounsaturated fatty acids (MUFAs) and polyunsaturated fatty acids (PUFAs) for *N. oculata* during nitrogen sufficient growth (N:P 20) and nitrogen limited growth (N:P 5 and N:P 2.5) after 4–5 days of N-limitation. N:P 20 shows mean value ± SD (*n* = 3), N:P 5 shows mean value ± SD (*n* = 2) and N:P 2.5 shows one value.

Accordingly, saturated fatty acids (SAFAs) increased during N-limited growth and together with monounsaturated fatty acids (MUFAs) constituted nearly 90% of total FA compared to 76% for the N:P 20 grown cultures (Figure 3). However, the SAFAs and MUFAs fraction during N-limitation was slightly higher in spring compared to autumn (Table 1). Similar levels for SAFAs and MUFAs were also shown by Rodolfi *et al.* [21] for *Nannochloropsis* sp. F&M-M24 strain at N-starvation. For this strain, Bondioli *et al.* [35] found more than 75% of SAFAs and MUFAs in N-starved culture and 70% of TL consisted of neutral lipids compared to 25% for control. SAFAs and MUFAs are mainly associated with neutral storage lipids in the form of triglycerides (TAGs), highly desirable in algal biofuels production [2,15,16]. Suen *et al.* [19] found 79% TAGs in N-deficient *Nannochloropsis* sp. QII. The fatty acids C16:0 and C16:1 were suggested to be the main storage lipids in *Nannochloropsis* sp. [11,29]. In the present study C16: 0, mainly, and C18:1 increased as a response to N-stress. Both these FA made up a large part of the SAFAs-MUFAs fraction (>86%). Previous studies [19,21,35] suggested that under nutrient stress some microalgae are able to store neutral oil compounds from *de novo* synthesized lipids without compromising the fraction or function of other lipid classes. Hence, the increase in SAFAs and MUFAs under N-limitation in our study was possibly allocated to the neutral lipid fraction.

**Table 1:** The major fatty acids (FAs) of the FA profiles (% of total FA) of *N. oculata* grown under nitrogen sufficiency (N:P 20) and nitrogen limitation (N:P 5 and N:P 2.5) in autumn 2008 and spring 2009. Profiles are shown for control and treatment at corresponding days: 0, 2, 5, 7, 8 in autumn 2008; 2, 4 during the first experiment in spring 2009; 4 during the second experiment in spring 2009.

**Autumn 2008**															
**Days**	**0**			**2**			**5**			**7**			**8**		
FA	N:P 20	N:P 5		N:P 20	N:P 5		N:P 20	N:P 5		N:P 20		N:P 5	N:P 20		N:P 5
C14:0	7.90	7.75		7.4	7.25		7.00	6.80		7.40		5.85	7.45		5.90
C16:0	39.80	39.40		36.75	43.30		31.00	39.75		40.50		43.30	39.80		43.95
C16:1	29.10	27.75		31.35	27.65		32.65	29.50		28.5		28.65	28.35		28.40
C18:1	5.35	7.20		5.20	7.15		4.50	8.30		5.20		8.45	3.80		8.80
C18:2	1.95	1.75		1.90	1.60		2.60	1.65		1.70		1.40	1.90		1.35
C20:4	4.30	3.60		4.75	2.95		6.60	3.65		4.35		3.10	5.00		2.90
C20:5	9.15	8.95		10.55	7.65		13.40	7.80		9.85		6.90	11.15		6.30
**Spring 2009**											**Spring 2009**				
**Days**	**2**					**4**					**4**				
FA	N:P 20		N:P 5			N:P 20			N:P 5		N:P 20			N:P 2.5	
C14:0	7.10		5.90			7.0			5.40		6.70			5.30	
C16:0	31.20		37.80			35.80			45.10		29.80			45.40	
C16:1	26.50		27.40			27.20			27.90		30.40			27.00	
C18:1	4.90		6.30			4.90			8.60		4.60			9.10	
C18:2	3.50		2.40			2.90			1.30		3.50			1.40	
C20:4	5.20		4.10			4.30			2.60		4.70			2.50	
C20:5	18.70		14.00			15.60			7.00		17.50			7.40	

Microalgae are also known to be a source of significant amount of polyunsaturated fatty acids (PUFAs) and *N. oculata* contained 11%–24% PUFAs of total FA. C20:5 (EPA) made up 6%–19% of total FA and decreased at N-limitation. High PUFAs content is not desirable according to the European standard for biodiesel [49] but can be mixed with other oils, hydrogenated or separated through fractional distillation. The average EPA content in the present study was approximately 13% at N-replete conditions and 8% at N-limited conditions and a rough estimation of the EPA productivity would then be around 13 mg L^−1^ day^−1^ for both strategies. EPA is a high value nutritional supplement (omega-3) for human and animal health benefits. Since EPA production can take place at both nutrient sufficient and nutrient limited growth, and if separated in an algal biorefinery, EPA should be considered as a major commercial co-product in algal biofuels production.

### 2.4. Biomass Productivity (BP) and Lipid Productivity (LP)

**Figure 4:**
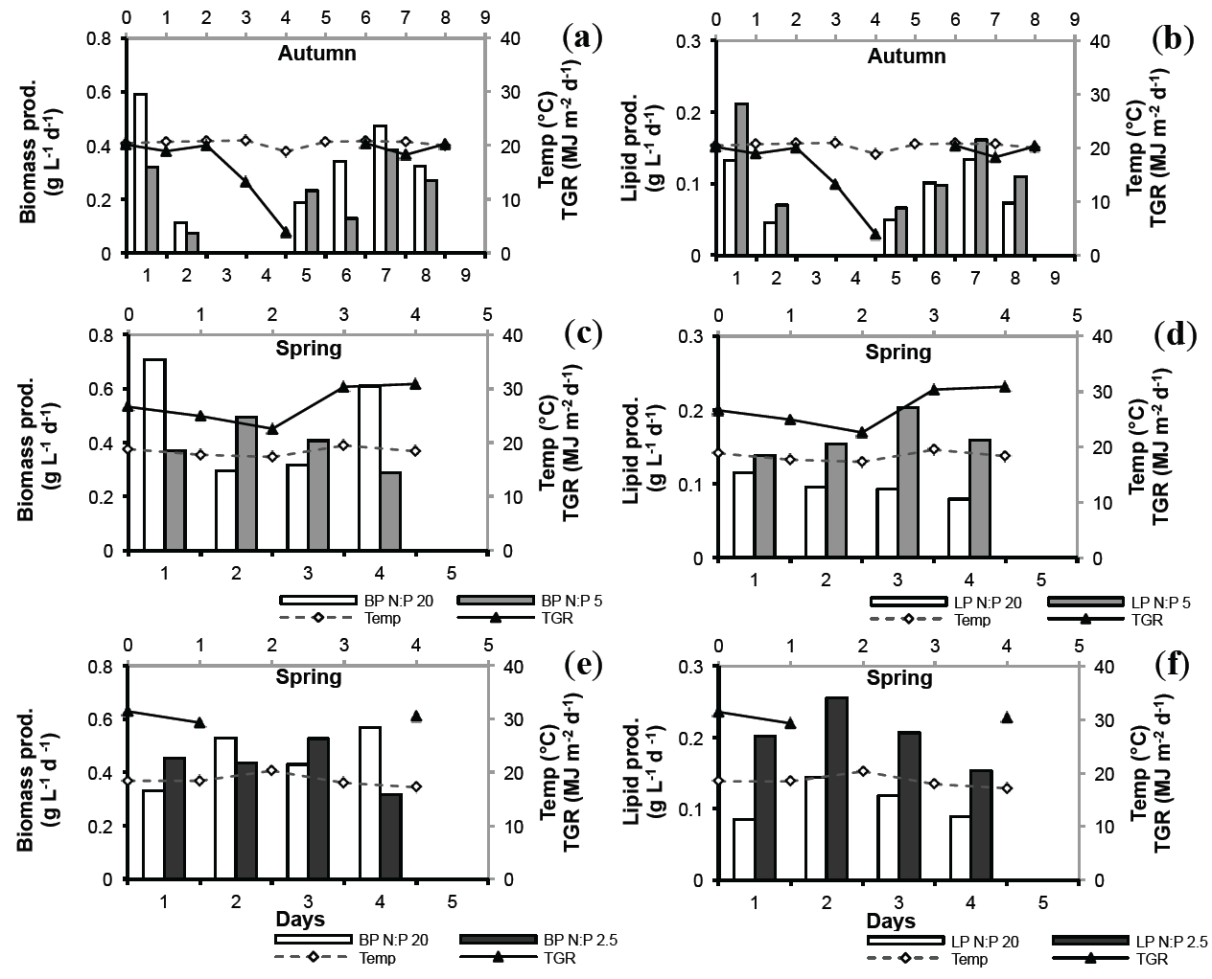
Biomass productivity (BP) and lipid productivity (LP), respectively, for *N. oculata* at; (**a**, **b**) N:P 5 in autumn; (**c**, **d**) N:P 5 in spring; and (**e**, **f**) N:P 2.5 in spring. BP is represented by white bars for controls (BP N:P 20) and medium grey bars for N-limitation (BP N:P 5, N:P 2.5). White bars represent control (N:P 20) for both BP and LP, light grey bars represent N:P 5 values for both BP and LP, and dark grey bars represent N:P 2.5 for both BP and LP. Temperature (open diamonds, dashed line) and total global radiation, TGR, (closed triangles, solid line).

The BP and LP over time for *N. oculata* during the different experiments are shown in Figure 4. Average values of BP and LP are presented in Table 2. The BP ranged 0.24–0.43 g L^−1^ day^−1^ for N-limited cultures and 0.34–0.48g L^−1^ day^−1^ for control cultures, with higher values in spring compared to autumn. No significant differences were found between N-limited cultures and controls (ANOVA, *p* = 0.7420) but with a minor significant seasonal effect (ANOVA, *p* = 0.0464). The level of N-stress in spring had no significant effect (ANOVA, *p* = 0.6145).

**Table 2:** Average biomass production (BP) and lipid production (LP) compared between N:P 20 (control) and treatments(N:P 5, N:P 2.5).

Nutrient Stress	Average BP (g L^−1^ day^−1^)		Average LP (g L^−1^ day^−1^)	
	Control	Treatment	Control	Treatment
N:P 5 autumn	0.34	0.24	0.09	0.12
N:P 5 spring	0.48	0.39	0.10	0.16
N:P 2.5 spring	0.47	0.43	0.11	0.21

For all three experiments the N-limited cultures produced significantly more lipids compared to control (ANOVA, N:P 5 autumn and spring: *p* = 0.0264; N:P 2.5 spring: *p* = 0.0051). At 15%–22% harvesting rate in autumn (Table 3) LP of the N:P 5 treatment (0.12 g L^−1^ day^−1^) was only slightly higher compared to N:P 20 (0.09 g L^−1^ day^−1^). In spring at 22% harvesting rate LP was 60% higher at N:P 5 (0.16 g L^−1^ day^−1^) compared to control (0.10 g L^−1^ day^−1^). More severe N-stress in spring (N:P 2.5) at 22%–33% harvesting rate resulted in 90% higher LP, 0.21 g L^−1^ day^−1^ compared to 0.11 g L^−1^ day^−1^ for the control. However, no significant seasonal effect was found (ANOVA, *p* = 0.1898).

**Table 3:** Nutrient ratios, concentrations and harvesting rate of the *N. oculata* cultures for treatments and control during autumn 2008 and spring 2009.

Parameter	N-sufficiency (control)	N-limitation	
N:P	20	5	2.5
Season	Autumn, Spring	Autumn, Spring	Spring
N	2000 µM	500 µM	250 µM
P	100 µM	100 µM	100 µM
Harvesting rate	15%–22%, 22%, 22%–33%	15%–22%, 22%	22%–33%

Clearly, nitrate limitation resulted in an increase of intracellular lipid content in *N. oculata* cultures*.* The present work also demonstrated a net gain in LP (up to 90%) for N-limited *N. oculata* cultures since relatively high biomass productivity (BP) was recorded for nutrient stressed cultures (N:P 2.5, during 5 days). Different ways to express productivity (volumetric and areal—illuminated surface or occupied ground) makes comparison among studies difficult, especially due to different PBR design. Two previous studies at up-scaled conditions expressing either volumetric or illuminated surface areal productivity [21,35] were compared to our result (Table 4). In these two studies, *Nannochloropsis* sp. F&M-M24 strain was grown in Green Wall Panel (GWP) PBRs (110 L and 590 L), applying N-starvation at 40% and 44% daily dilution rate, respectively. Normalizing productivity values to volumetric productivity, Table 4 shows that BP and LP in the present study, equated well to these previous studies using *Nannochloropsis* at up-scaled conditions [21,35].

Differences in lipid contents and productivities may be due to strain specificity but could also be an effect of cultivation approach (N-starvation or N-limitation) and dilution rate. In order to optimize lipid production in large-scale cultures of microalgae, further fine-tuning of growth conditions and nutrient stress level is required. Possibly the higher harvesting rate during N:P 2.5 growth (22%–30%) compared to N:P 5 growth (22%) also had an effect on the productivity attributed to a more diluted culture with higher light capturing efficiency. On the other hand, if the harvesting rate is too great the culture will be too diluted to maintain a stable productivity. Empirical evidence suggested that maximum culture productivity is attained at dilution rates approximately half the maximum specific growth rate [50]. Applying a harvesting rate of at least 30% but probably not more than 40% would then yield in a net gain in LP of approximately 30%–100% compared to N-sufficiently grown cultures. For this particular strain a similar cultivation approach to that suggested by Rodolfi *et al.* [21], where cultures are grown in sufficient growth medium to high cell density before N-limitation is induced, can be proposed.

**Table 4:** A comparison among the present study, Rodolfi *et al.* [21] and Bondioli *et al.* [35] concerning the variables biomass productivity (BP) and lipid productivity (LP) expressed as volumetric productivity (g L^−1^ day^−1^) at N-sufficiency, N-limitation and N-starvation (n.a. = not available). * The values from Bondioli *et al.* [35], originally given as g m^−2^ of illuminated reactor surface day^−1^, were normalized to volumetric productivity.

Study	Dilution Rate	PBR Volume (L)	Variable	N-sufficiency (g L^−1^ day^−1^)	N-limitation (g L^−1^ day^−1^)	N-starvation (g L^−1^ day^−1^)
Present study	30%–33%	1374	BP	0.48	0.43	n.a.
			LP	0.11	0.21	n.a.
Rodolfi *et al.* [21]	40%	110	BP	0.36	0.22	0.30
			LP	0.12	0.11	0.20
Bondioli *et al.* [33]	44%	590	BP	n.a.	n.a.	0.33 *
			LP	n.a.	n.a.	0.22 *

Table 5 shows projections of biomass and lipid yields. Based on our results, LP of 0.21 g L^−1^ day^−1^, considered as a best-case scenario, would at a full growth season (350 days) eventuate a total lipid yield of 13 t ha^−1^ year^−1^. A more cautious calculation based on an annual average LP of 0.10–0.15 g L^−1^ day^−1^ would project an annual lipid yield of 8.0–10 t ha^−1^ year^−1^ at N-limitation (Table 4). Maintaining LP of 0.21 g L^−1^ day^−1^ on annual basis will be difficult. Hence, the cautious scenario may be a more realistic baseline but with potential of attaining higher yield from optimization and extensive R&D.

**Table 5:** Biomass productivity (BP), lipid productivity (LP), biomass and lipid yield predictions for a flow-through flat panel PBR system cultivating *N. oculata* in the south of Portugal. Projections are based on experimental data (this study, Olofsson *et al.* [26]), a PBR volume of 1374 L, an occupied ground area of 75 m^2^ per PBR and a growth season of 350 days.

Treatment	Cautious Scenario		Best-Case Scenario	
	Volumetric (g L^−1^ day^−1^)	Annual Yield (t ha^−1^ year^−1^)	Volumetric (g L^−1^ day^−1^)	Annual Yield (t ha^−1^ year^−1^)
*BP*				
N-sufficiency	0.30–0.40	19–26	0.40–0.50	26–32
N-limitation	0.25–0.35	16–22	0.35–0.45	22–29
*LP*				
N-sufficiency	0.05–0.10	3.0–6.0	0.10–0.12	6.0–8.0
N-limitation	0.12–0.15	8.0–10	0.15–0.20	10–13

### 2.5. Lab vs. Large Scale

Numerous lab-based studies reported BP and LP of different *Nannochloropsis* strains ranging 0.2–2.9 g L^−1^ day^−1^ [13,34,41,42,47,51] and 0.02–0.48 g L^−1^ day^−1^ [24,34,41,42,52,53,54], respectively. Our results are in the lower range regarding BP (0.24–0.47 g L^−1^ day^−1^) and in the middle range concerning LP (0.08–0.21 g L^−1^ day^−1^). Different culture conditions within these studies complicate comparison. Furthermore, few studies documented BP and LP from outdoor large-scale experiments. Zittelli *et al.* [12] investigated eicosapentaenoic acid (EPA) production of *Nannochloropsis* sp. in outdoor tubular reactors (610 L) in Florence, Italy. In September, BP ranged 0.57–0.73 g L^−1^ day^−1^ during two consecutive years was, while in May BP reached 0.76 g L^−1^ day^−1^. Considering the FA content (13%–21%), LP ranging 0.07–0.16 g L^−1^ day^−1^ would be possible, with average EPA productivity (5 months) of 24 mg L^−1^ day^−1^ [12] to compare with 13 mg L^−1^ day^−1^ for our experiments. The present study would end up in the lower range concerning BP, both spring and autumn, but in the same range concerning LP but with less EPA productivity. As shown in Table 4, our results are in the same range for both BP and LP of other outdoor up-scaled studies with *Nannochloropsis*.

High LP was reported for other species at lab scale under different culture conditions, ranging 0.04–0.46 g L^−1^ day^−1^ [37,41,42,55,56,57,58,59]. More relevant and interesting for future studies would be long-term results on annual basis from outdoor large-scale operated plants monitoring BP and LP from applied nutrient manipulation. As a rare report, Moheimani and Borowitzka [60] found the haptophyte *Pleurochrysis carterae* to attain a decent annual average BP of 22 g m^−2^ day^−1^ in outdoor raceway ponds at nutrient replete conditions. At an outdoor algae production facility (total capacity 174,000 L) consisting of panel photobioreactors submerged in water basins, Quinn *et al.* [61] reported annual average biomass productivity for *N. oculata* from May 2008 to June 2009 and *N*. *salina* from April 2009 to January 2011 (0.15–0.16 g L^−1^ day^−1^) with peak values of 0.37 g L^−1^ day^−1^. Average lipid yield reached approximately 7–13 m^3^ ha^−1^ year^−1^ (approximately 6.5–10 t ha^−1^ year^−1^) with peak values of 36 m^3^ ha^−1^ year^−1^ (33 t ha^−1^ year^−1^).

Extrapolations of lab-based, indoor or small-scale data to outdoor mass-cultivation of microalgal oil yield up to 80–130 t ha^−1^ year^−1^ [1,2,62] seem unrealistic. Weyer *et al.* [63] estimated the annual oil yield for a best-case scenario to be 37–49 t ha^−1^. However, Rodolfi *et al.* [21], obtaining the same lipid productivity as in our experiment, estimated oil yield, at a best-case scenario, to be about 20 t ha^−1^ year^−1^ for a production plant constructed of cheap plastic green wall panels (GWP) in Tuscany, Italy and up to 30 t ha^−1^ year^−1^ was suggested for tropical regions. Table 5 depicts both a cautious and a best-case scenario based on our results. The present work was conducted with flow-through flat panel PBRs in the south of Portugal, receiving slightly higher annual solar radiation than Tuscany. Higher BP compensated the lower lipid content in the *N. oculata* strain used in the present study. Our results demonstrated that different PBR design and formation of algae production units might result in different volume to areal footprint efficiency and thus different areal oil yield (Table 5), even though volumetric productivity is similar. Nevertheless, it also shows the potential for the Necton *N. oculata* strain, in the south of Portugal at a best-case scenario, to attain oil yields of 20 t ha^−1^ year^−1^. Our scenarios (Table 5) show that seasonal variation may result in lower estimates of the annual lipid productivity compared to the literature, and emphasize the importance of including seasonal variation in annual economic projections.

### 2.6. Seasonal Variation

**Figure 5:**
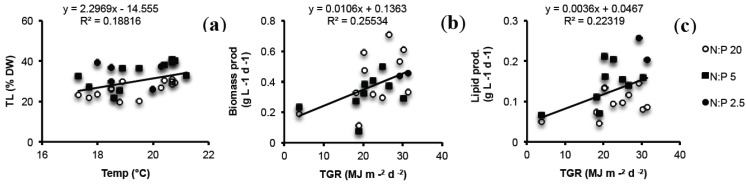
(**a)** Total lipids (TL) as a function of temperature for all nutrient manipulation experiments; (**b**) biomass productivity; and (**c**) lipid productivity as a function of total global radiation (TGR) for all nutrient manipulation experiments. Regression lines disclose the relationship of the complete datasets for respective variable (simple regressions, TL: *p* = 0.0104, BP: *p* = 0.0164 and LP: *p* = 0.0264).

Lipid content and composition have previously been shown to vary with season in this *N. oculata* strain [26]. In the present study, the initial lipid content was higher in autumn than in spring in accordance to Olofsson *et al.* [26]. A positive significant relationship was also found between TL (all experiments) and temperature explaining 19% of the variation (simple regression, *p* = 0.0104) as shown in Figure 5. Even though the differences in lipid content between treatment and control seemed not to be affected by the degree of N-limitation, applying N:P 2.5 stress in summer-autumn when lipid content in general is high, could possibly lead to a pronounced increase in TL and consequently also boost LP. On the other hand, TP values were similar comparing autumn and spring experiments.

Although temperature explained 19% of the variation in TL, no significant effects of temperature on BP and LP was found (simple regressions, *p* > 0.05). Positive significant relationships were found between both BP and LP and TGR explaining 26% and 22% (simple regressions, *p* = 0.0164 and *p* = 0.0264) of the variation, respectively (Figure 5). Consequently, slightly higher BP was observed in spring compared to autumn in both treatments and controls. The higher BP in spring also resulted in enhanced LP in spite of lower TL content. Therefore, seasonal variations of light and temperature need to be considered, in combination with nutrient manipulation, when projecting oil yield of microalgae. Nonetheless, the results suggested a major role for N-stress as such.

Lipid composition of *N. oculata* changed with N-stress. Increasing the stress from N:P 5 to N:P 2.5 did not further change lipid quality. No major change in lipid composition for the N-limited cultures could be found when comparing autumn *vs.* spring experiments. However, for control cultures especially C16:0 was higher and C20:5 was lower in autumn compared to spring, which was suggested to be an effect of changing light and temperature [26].

Our results may also have implications for molecular engineering. Starchless mutant strains of the freshwater green algae *Chlamydomonas* was found to accumulate up to 65% lipids under N-deficiency compared to 13% for wild type strains [64]. Typically, the vast majority of engineered efforts have been done on *Chlamydomonas*. However, the more widely available molecular techniques may in a near future facilitate metabolic engineering of other wild type algal strains that accumulate oil as a natural response to N-limitation. In addition, recent improvements of multispecies microbial cell factories represent an interesting approach [65]. Genetically engineered *Nannochloropsis*-bacteria mutualistic relationships providing carbon and nutrient recycling could possibly enhance lipid production and other high value products even more.

## 3. Experimental Section

### 3.1. Photobioreactors (PBRs) and Experimental Set Up

*N. oculata* (commercial strain, Necton) was inoculated and grown in batch mode outdoors in two adjacent closed flow-through vertical flat panel PBRs (1374 L) at Necton’s facility in Algarve (Olhão, Portugal). The water used for cultivation was pumped from ground seawater (salinity 35‰), filtered first through a 5 µm cartridge filter followed by hypochlorite addition in a disinfection tank. Before a second cartridge filtration (1 µm) hypochlorite was neutralized with tiosulphate.). Nutrients were added as NutriBloom medium (Necton’s commercial and industrial culture medium recipe) to reach nitrate levels of 2 mM and phosphate levels of 0.1 mM (final concentration in circulating cultures, N:P = 20). Original NutriBloom medium contains 2 M NaNO_3_, 100 mM KH2PO_4_, 20 mM FeCl_3_, 20 mM EDTA-Na, 1 mM ZnCl_2_, 1 mM ZnSO_4_-H_2_O, 1 mM MnCl_2_·4H_2_O, 0.1 mM Na_2_MoO_4_·2H_2_O 0.1 mM CoCl_2_·6H_2_O, 0.1 mM CuSO_4_·5H_2_O, 6.4 mM EDTA-Na, 2 mM MgSO_4_-7H_2_O. Nitrate concentrations were monitored daily (data not shown) and fresh NutriBloom medium was added to maintain nitrate concentration at 2 ± 0.2 mM and constant N:P = 20 to ensure sufficient nutrient availability. When the online pH set point reached 8.5, CO_2_ was injected into the system at 2 bar pressure. The pH ranged 7.5–9.5 during the day but could drop to as low as 6.0 at night. When culture temperature in the PBRs reached 25 °C the reactor panels were cooled with water sprinklers. The temperature varied from 10 °C at night to 30 °C during the day despite cooling of the panels.

In autumn 2008 (24 September–2 October) and spring 2009 (11–15 May and 18–22 May) nitrogen levels were manipulated in large-scale cultures of *N. oculata* in the south of Portugal (Olhaõ). *N. oculata* was grown in semi-continuous mode under nutrient repletion (N:P 20) and nitrogen limitation (N:P 5, N:P 2.5) according to Table 3. PBRs were operated with a daily harvesting rate of 22%–33% and replacement of the culture volume with fresh medium at different N-levels (Table 3). Each N-level treatment was run concurrently with a control (N:P 20). The experiments were run for 9 days (autumn) and 5 days (spring) as the autumn experiment showed a rapid response (24 h) to N-stress. Daily sampling included nitrate concentrations in the N-limited treatments (N:P 5, N:P 2.5), OD, DW and TL. Samples for TP and FA profiles were collected every second day.

### 3.2. Analyses Methods

Samples for nitrate analysis were collected before harvest and before replenishment of new medium. Nitrate concentrations were determined according to Eaton *et al.* [66], by centrifuging 8 mL of *N. oculata* culture (2000× *g*) and adding 1 mL of the supernatant to 8.8 mL of NaCl solution (35 g L^−1^) followed by addition of 0.2 mL of 1 M HCl and measuring the absorbance at 220 nm and corrected for organic matter at 275 nm in the spectrophotometer. OD was measured in the spectrophotometer at 540 nm as a proxy for biomass. DW was determined by filtering 5–10 mL of algal culture onto pre-weighed 45 mm glass fiber filters, rinsed with 10 mL ammonium formiate (0.5 M) and dried in the oven at 70 °C until constant weight.

TL was determined according to Bligh & Dyer [67], modified as following: 30–50 mL of culture were centrifuged for 15 min (2000× *g*), the supernatant was removed and the algal pellet dissolved in chloroform:methanol (1:2 v/v) mixture followed by sonication for 5 min. Samples were put in the fridge over night followed by centrifugation for 5 min (2000× *g*) and collection of the supernatant. This extraction procedure was repeated 2–3 times for full extraction since *Nannochloropsis* cells have a rigid cell wall difficult to rupture. To the collected supernatant chloroform and distilled H_2_O were added to a final ratio of 2:2:1 chloroform:methanol:H_2_O v/v and centrifuged for 5 min (2000× *g*) to separate the lipid phase in chloroform from the water-methanol phase. The latter phase was discarded and the chloroform-lipid phase was transferred to pre-weighed glass tubes. Chloroform was evaporated in the oven at 55 °C until constant weight.

TP were determined according to the method of Lowry *et al.* [68] modified by Herbert *et al.* [69]. In brief, 10 mL of algal culture were centrifuged (2000× *g*), the supernatant discarded and the pellet resuspended in 2 mL 1 N NaOH and put in a water bath at 95–100 °C for 60 min. Samples were cooled down in room temperature and centrifuged (2000× *g*) for 10 min. 100 µL of the supernatant was transferred to a new glass tube and mixed with 300 µL dH_2_O and 400 µL 1 N NaOH. 2 mL of freshly prepared Reagent A (50 mL 5% NaCO_3_ + 2 mL (0.5% CuSO_4_·H_2_O + 1% potassium sodium tartrate)) was added to the samples and agitated. After 10 min in room temperature 400 µL of freshly prepared Reagent B (1:1 Folin-Ciocalteau:dH_2_O) was added to the samples and mixed. The samples were incubated for 30 min in room temperature. Absorbance was measured at 750 nm in the spectrophotometer and compared to a standard curve prepared from bovine serum albumin (20–300 µg mL^−1^).

For FA 1 L of algal culture was centrifuged for 15 min at 7520× *g* (Beckman Avanti™ J-25, Beckman Coulter, Inc., Brea, CA, USA), and algal paste frozen at −20° C. Rests of seawater salt was removed from the paste according to Olofsson *et al.* [26]. Algal paste was stored frozen (−20 °C) prior to freeze-drying. FA profiles were determined using gas chromatography (GC) at Ifremer, Nantes, France [70]. An aliquot of lipid was evaporated under nitrogen and trans-methylated by contact with methanol-sulfuric acid (98:2, v/v) in excess at 50 °C overnight. After cooling, 2 mL of hexane and 1 mL of water were added and vortexed. The upper organic phase containing fatty acid methyl esters (FAMEs) was collected and assayed by gas chromatography using a PerkinElmer Auto system equipped with an FID detector. Separation was done using helium as carrier gas on a fused-silica column (BPX-70, 60 m long, 0.25 mm i.d., 0.25 μm film thickness, SGE Analytical Science Pty Ltd., Ringwood, Australia) programmed from 55 °C (for 2 min) to 150 °C at 20 °C min^−1^ then to 230 °C at 1.5 °C min^−1^. Sample was injected with a programmable split/splitless inlet and large-volume injection system (PSS) using the following temperature program: 55 °C (for 2 min) to 350 °C at 200 °C min^−1^. FAMEs were identified by comparison of their equivalent chain length with those of authentic standards. Quantification was done using margaric acid (C17:0) as internal standard.

Biomass and lipid productivity were determined from the difference in DW or TL, both in g L^−1^, between two sampling days taking into account the remaining biomass or lipid content after harvest. Biomass productivity:

BP = DW_t2_ − (Remaining Biomass (%) × DW_t1_) (1)

where BP is biomass productivity, DW_t2_ is the dry weight at a specific day, DW_t1_is the dry weight at the previous day and Remaining Biomass is the percentage of biomass remaining after harvest (e.g., 80% at a harvesting rate of 20%).

Lipid productivity:

LP = TL_t2_ − (Remaining Biomass (%) × TL_t1_) (2)

where LP is lipid productivity, TL_t2_ is the total lipids (g L^−1^) at a specific day, TL_t1_ is the total lipids at the previous day and Remaining Biomass is the percentage of biomass remaining after harvest (e.g., 80% at a harvesting rate of 20%). Biomass and lipid productivity are expressed in g L^−1^ day^−1^.

Projections of biomass and lipid yields in Table 4 were based on the PBR volume (1374 L) and the occupied ground area, including ancillary equipment (75 m^2^).

Temperature data was obtained from the meteorology portal Clima Tiempo Meteored [71] and light in the form of total global radiation (TGR) was obtained from the Meteorological Institute of Portugal (Instituto Português do Mar e da Atmosfera).

To test the effect of N-limitation and season on the response variables (DW, TL, TP, BP, LP), we started with a linear model including the time effect as a continuous covariate (ANCOVA). By backward stepwise procedure in R (version 3.0.2, R Foundation for Statistical Computing, Vienna, Austria) the simplest model with the highest explanatory power was applied to each variable. The same procedure was used to test the effects of three different levels of N-limitation (N:P 20, N:P 5, N:P 2.5) on the response variables in spring. Assumptions of normality and homogeneity of variances were checked by plotting residuals *versus* predicted values. Simple regressions between TL, BP and LP as the dependent variables and temperature and TGR as the independent variables were performed using GraphPad Prism 6 (GraphPad Software Inc., La Jolla, CA, USA).

## 4. Conclusions

The present study shows under commercially realistic conditions (outdoor, different seasons) that *N. oculata* is a suitable candidate in biofuel production since BP was maintained at a high level under N-limitation resulting in a total net gain in LP. Dilution rate optimization during nutrient manipulation needs to account for seasonal variation of biomass and lipid productivity and adjusted accordingly. Despite the 50-year-old concept of algae based biofuel, no commercially viable production is yet in use. In terms of production capacity, to meet promising predictions based on lab work, the only solution would be scaling up, producing biomass and algae oil on annual basis, utilizing cheap nutrients and CO_2_ from exhaust/waste streams. Sustainability challenges must be met by recycling of water and nutrients. Industrial waste products have to be seen as resources in order to produce a wide range of high value components from microalgae including sustainable fuel production. Closed PBRs may be used to produce fine chemicals (PUFAs, peptides, pigments, *etc.*) with high revenue potential to finance R&D efforts into algae based biofuels. As shown in the present study, EPA production may be feasible at both N-sufficiency and serve as a valuable co-product in biofuel production at N-limitation. The PBRs also produce large enough inoculum for open raceways where algal cultures convert cheap waste streams and exhaust gases to bulk chemicals for fuel and industrial material with the aid of N-stress to boost lipid production.
